# Functional Connectivity Disruptions in Grey Matter Volume‐Altered Brain Regions Among Male Smokers: A Neuroimaging Study

**DOI:** 10.1111/adb.70096

**Published:** 2025-11-28

**Authors:** Hui Zhang, Jiawen Tian, Xinyu Wang, Hongyu Zhang, Longyao Ma, Bohui Mei, Mengzhe Zhang, Qingqing Lv, Yarui Wei, Shaoqiang Han, Yong Zhang

**Affiliations:** ^1^ Department of Magnetic Resonance Imaging The First Affiliated Hospital of Zhengzhou University Zhengzhou Henan China; ^2^ Department of Radiology The Third Affiliated Hospital of Zhengzhou University Zhengzhou Henan China

**Keywords:** cigarette smoking, executive control network (ECN), functional connectivity (FC), grey matter volume (GMV), superior frontal gyrus

## Abstract

This study aimed to investigate whether structural brain alterations in smokers are accompanied by synchronized changes in functional connectivity, with a focus on understanding the neural mechanisms underlying smoking addiction. We conducted a meta‐analysis of voxel‐based morphometry (VBM) studies using the Seed Mapping software package to identify consistent grey matter changes in smokers' brains, which were subsequently defined as regions of interest (ROIs). Resting‐state functional connectivity between these ROIs and whole‐brain voxels was analysed in 53 male smokers and 38 non‐smokers. Additional correlation analyses were performed to assess relationships with clinical features. Smokers exhibited reduced functional connectivity between the right lingual gyrus (which showed increased grey matter volume) and the left calcarine sulcus. The right superior frontal gyrus (with decreased grey matter volume) suggested diminished functional connectivity with multiple regions, including the bilateral precentral and postcentral gyrus, left rolandic operculum, left inferior frontal gyrus (opercular part), left midcingulate cortex, bilateral supplementary motor area, left paracentral lobule, right inferior frontal gyrus (triangular part) and right middle frontal gyrus (GRF corrected, voxel‐level *p* < 0.001, cluster‐level *p* < 0.05). Notably, reduced connectivity of some brain regions to the right superior frontal gyrus was negatively correlated with the Fagerström Test for Nicotine Dependence (FTND). Our findings demonstrate that structural brain alterations in smokers are associated with specific disruptions in functional connectivity, particularly within visual attention, executive control and sensorimotor networks. These results provide additional evidence of the neuropathophysiological mechanisms underlying smoking addiction.

## Graphical abstract

Based on a meta‐analysis of gray matter structural alterations in smokers and functional connectivity analysis, disrupted functional connectivity was observed in certain brain regions exhibiting structural changes, particularly within the visual attention, executive control, and sensorimotor networks. This finding provides new insights into the neuropathophysiological mechanisms underlying nicotine addiction.

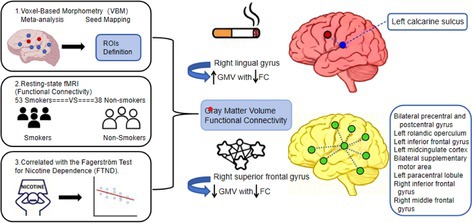

## 1 Introduction

The tobacco epidemic is one of the greatest public health challenges the world has ever faced, killing more than 8 million people worldwide each year [1]. Some studies have shown that most of the increase in mortality is due to tumour, vascular, respiratory and other diseases caused by smoking [2]. Although the dangers of smoking are well known, only about one in five smokers succeed in quitting with the help of medication [3]. Therefore, it is important to understand how tobacco alters brain structure and function that may help guide smoking cessation. Voxel‐based morphometry (VBM) is a neuroimaging method used to study structural differences in the brain, allowing comparison of differences in brain structure across individuals [4]. With VBM, researchers have found in previous studies that smoking has an effect on brain structure, with smokers experiencing changes in grey matter volume or density in different brain regions compared to non‐smokers. Some studies have found that smokers have decreased grey matter volume in brain regions, such as the thalamus, putamen, precuneus and cingulate gyrus [5, 6, 7, 8, 9], and increased grey matter volume in the left middle occipital gyrus and right lingual gyrus [10]. Brain structure is the basis of brain function and behaviour, and the integrity of brain structure is closely related to changes in brain function [11]. Seed‐based functional connectivity, also known as region of interest (ROI)–based functional connectivity, is able to compute interrelationships between seeds and time series of other parts of the brain [12]. In this study, a meta‐analysis of the found smokers' VBM studies was performed using the Seed‐based d Mapping (SDM) package to obtain brain regions with consistent structural brain changes in smokers. Seed‐based functional connectivity of these brain regions was performed, followed by a correlation analysis of the areas of differential brain activity with the clinical characteristics of smokers. The aim of this study was to find out whether there are corresponding alterations in the functional connectivity of the brain in the structurally altered brain regions of smokers and the possible relationship between the altered functional connectivity and the clinical characteristics of smokers.

## 2 Materials and Methods

### 2.1 Meta‐Analysis

#### 2.1.1 Literature Search and Selection

In the PubMed, Web of Science and Scopus databases, using a few keywords (‘Voxel‐based morphometry’ or ‘VBM’ or ‘grey matter’) and (‘smoking’ or ‘nicotine’ or ‘tobacco’ or ‘cigarette’ or ‘smokers’), a comprehensive and exhaustive search of VBM studies of smoking from January 2010 through December 2024 was conducted. At the same time, references related to high‐quality and relevant meta‐analyses and reviews were searched to avoid omissions [13].

Studies were included if the following inclusion criteria were satisfied: (1) The patient group included chronic cigarette smokers without other diseases such as Parkinson's disease (PD), multiple sclerosis (MS) and so on; (2) VBM method was used in the comparison of brain GM changes between chronic smokers and non‐smokers; (3) peak coordinates from the Montreal Neurological Institute (MNI) or Talairach space were provided; (4) whole‐brain results were reported and consistent thresholds were used across brain regions. Studies were excluded if (1) they only reported region of interest findings; (2) no VBM was used; (3) peak coordinates were not reported and the authors could not be contacted to obtain them; (4) inconsistent thresholds were used in different brain regions; or (5) no corresponding healthy control group.

**FIGURE 1:**
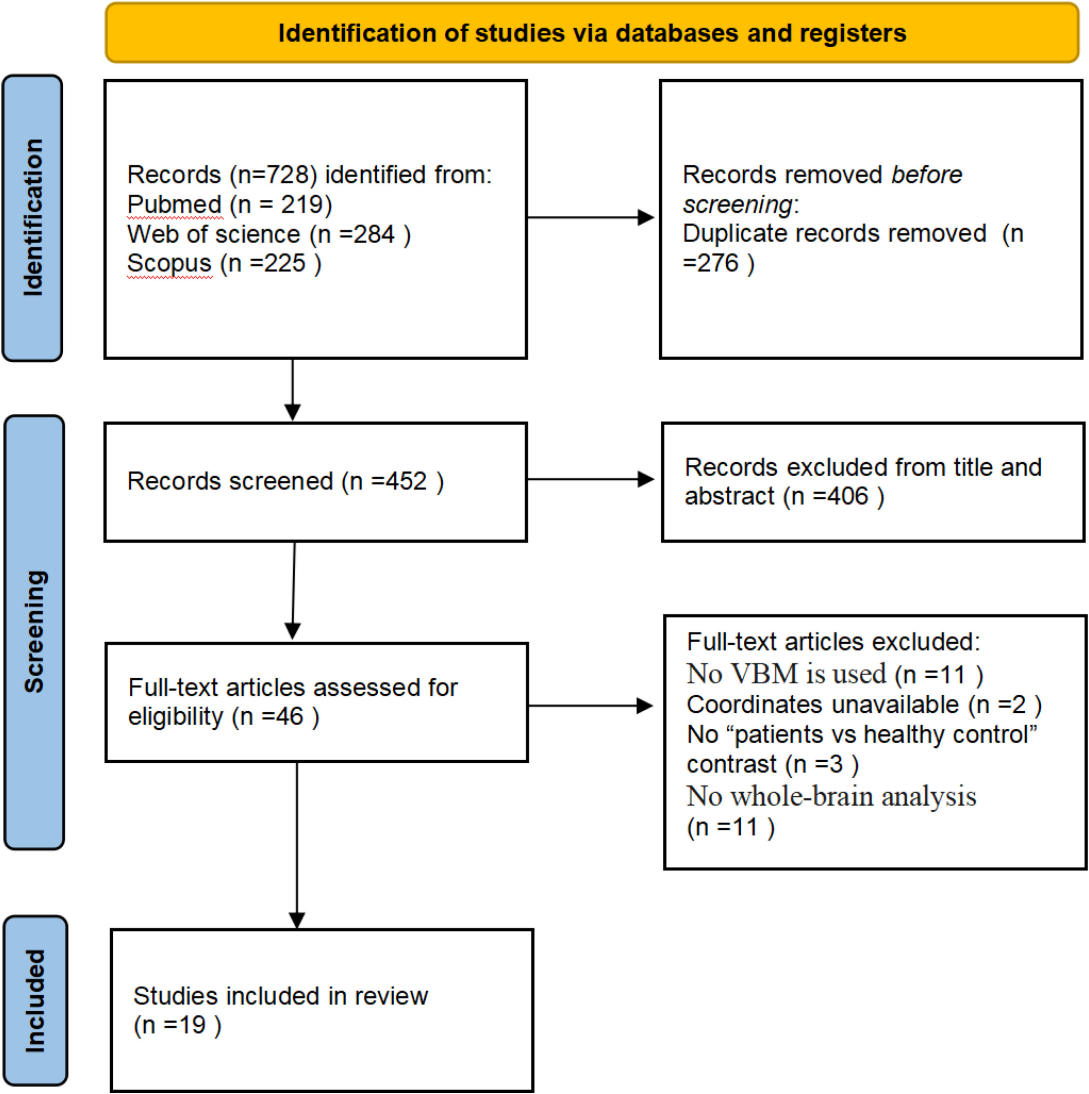
Search Strategy used for the inclusion of studies that were considered for the meta‐analysis.

Two authors independently searched, selected and cross‐checked the literature. Disagreements were resolved through a joint reassessment of the original studies, after which a consensus decision was reached and the following steps were implemented. This study followed the Preferred Reporting Items for Systematic Evaluation and Meta‐Analysis (PRISMA) guidelines [14] (Figure 1).

#### 2.1.2 Statistical Analysis

We used the anisotropic effect size version of the SDM package (Version 5.15) to perform the meta‐analyses. SDM has been extensively validated and well illustrated in several studies in the field of neurology [15, 16]. Three‐dimensional peak coordinates and effect sizes (*t* values) in MNI space from the VBM studies were extracted according to the AES‐SDM guidelines, which utilize an anisotropic non‐normalized Gaussian kernel to recreate voxel level maps for each study [17, 18]. The steps are as follows: (1) Use the icbm2tal (Lancaster) transform to convert the coordinates reported in Talairach space to MNI coordinates [19]. (2) Some studies did not have *t* values and converted *p* values or *z* values to *t* values via an online converter. (3) If there is no effect size (*t* value, *z* value, *p* value or similar), we use ‘p’ for positive peaks and ‘n’ for negative peaks. (4) The full width at half peak (FWHM) was set to 20 mm, as this would maintain a balance between sensitivity and specificity, as well as other parameters including voxel *p* < 0.005 (peak height threshold > 1, clustering range threshold > 10 voxels). (5) After removing one study at a time, the meta‐analysis was repeated for folding knife sensitivity analysis to verify the stability and reliability of the results [16].

### 2.2 Functional Connectivity

#### 2.2.1 Participants

The study included 97 male participants (57 smokers and 40 non‐smokers) aged 20–55 years who were recruited from local hospitals or via the Internet. Six smokers were excluded from preprocessing, and the participants in the follow‐up functional connectivity study were 53 smokers and 38 non‐smokers. Because the prevalence of smoking is much higher in Chinese men than in women, and the burden of public health falls mainly on this group, this study examines male smokers [20]. Inclusion criteria for the smoking group: (1) right‐handedness; (2) meeting diagnostic criteria for substance dependence in the fifth edition of the Diagnostic and Statistical Manual for Mental Disorders (DSM‐5); and (3) smokers who have smoked for at least 2 years and at least ten cigarettes per day and have quit no more than 3 months ago [21]. Non‐smokers should smoke fewer than five cigarettes in their lifetime [22]. Non‐smokers were matched to the smoker group for age, gender and education. Exclusion criteria for participants included the presence of addiction to substances other than nicotine; the presence of neuropsychiatric disorders; contraindications to MRI; and physical illnesses, including obstructive lung disease, epilepsy, brain tumours or cerebrovascular disease [23]. We used the Fagerström Test for Nicotine Dependence (FTND) to measure the severity of tobacco addiction and collect clinical information related to smoking, such as smoking age and daily cigarette consumption. Pack‐years are calculated as (years of smoking multiplied by number of cigarettes smoked per day)/20 [24]. The trial was approved by the Medical Ethics Committee of the First Affiliated Hospital of Zhengzhou University, and informed consent was obtained from all subjects.

#### 2.2.2 MRI Data Acquisition

MRI data were acquired using a 3‐T Siemens MAGNETOM Skyra scanner equipped with a 16‐channel head coil. Smokers were asked to smoke 30 min before the scan to avoid nicotine withdrawal symptoms. During the scanning process, each participant was asked to close their eyes to stay awake and avoid falling asleep, and participants who fell asleep during the process were eliminated. We used foam pads to immobilize the head to minimize the effects of head movements. The parameters of the images were as follows: repetition time (TR)/echo time = 2000/30 ms, matrix size = 64 × 64, flip angle = 80°, field of view = 240 × 240 mm, voxel size = 3 mm × 3 mm × 3 mm, slices = 36, slice thickness = 4 mm and a total of 180 volumes.

#### 2.2.3 Data Analysis

Preprocessing of functional imaging data uses the Data Processing and Analysis for Brain Imaging (DPARSF) toolbox. The main preprocessing steps and parameters were as follows: (1) conversion of DICOM images to NIfTI files; (2) deletion of the first five time points to minimize the effect of initial signal instability in rs‐fMRI; (3) slice timing; (4) realignment (exclusion of subjects with maximal head movement > 2.5 mm or head rotation > 2.5°. Six participants were excluded [four smokers and two non‐smokers]); (5) normalization using the EPI template and resampling using a voxel size of 3 × 3 × 3 mm; (6) linear trending and temporal filtering (bandpass, 0.01–0.08 Hz) to remove low‐frequency drift and high‐frequency noise effects; (7) covariate regressions were performed on 24 head motion parameters, cerebrospinal fluid signals and white signals, with global signal regressions omitted due to related controversies [25, 26]; (8) frame‐by‐frame displacements (FDs) were computed for each time point [27], and average FDs greater than 0.5 mm were excluded; and (9) a 6‐mm full‐width‐half‐maximum (FWHM) Gaussian kernel was used for smoothing.

A method based on ROI seeding was applied to assess rs‐FC differences between smokers and non‐smokers. By meta‐analysis, brain regions with significant differences in brain structure between smokers and non‐smokers were used as ROIs, which were defined as spheres with a radius of 5 mm centred on the peak coordinates. Pearson's correlation coefficients were calculated between the mean time series of the seed regions and the time series of all voxels in the whole brain to derive rs‐FC plots for each subject. The correlation coefficients were converted to Z‐scores by the Fischer Z‐transform to improve data normality.

#### 2.2.4 Statistical Analyses

Clinical data (e.g., age and years of education) were analysed using two independent samples *t*‐tests and non‐parametric tests for normally and non‐normally distributed data, respectively. Age, years of education and mean FD were used as covariates in a two‐tailed two‐sample *t*‐test based on the MATLABSPM 12 toolkit to compare the rs‐FC based on structurally differential brain regions between the two groups (GRF correction: P voxels < 0.001, P clustering < 0.05). To investigate the relationship between brain regions with differential functional connectivity and clinical characteristics of smoking, we used Spearman's correlations [28] to measure the correlation between FC values of brain regions with differential functional connectivity and FTND scores, age at smoking, number of cigarettes smoked and peak‐years.

## 3 Result

### 3.1 Meta‐Analysis of Pooled GM Difference Brain Region Results

**FIGURE 2:**
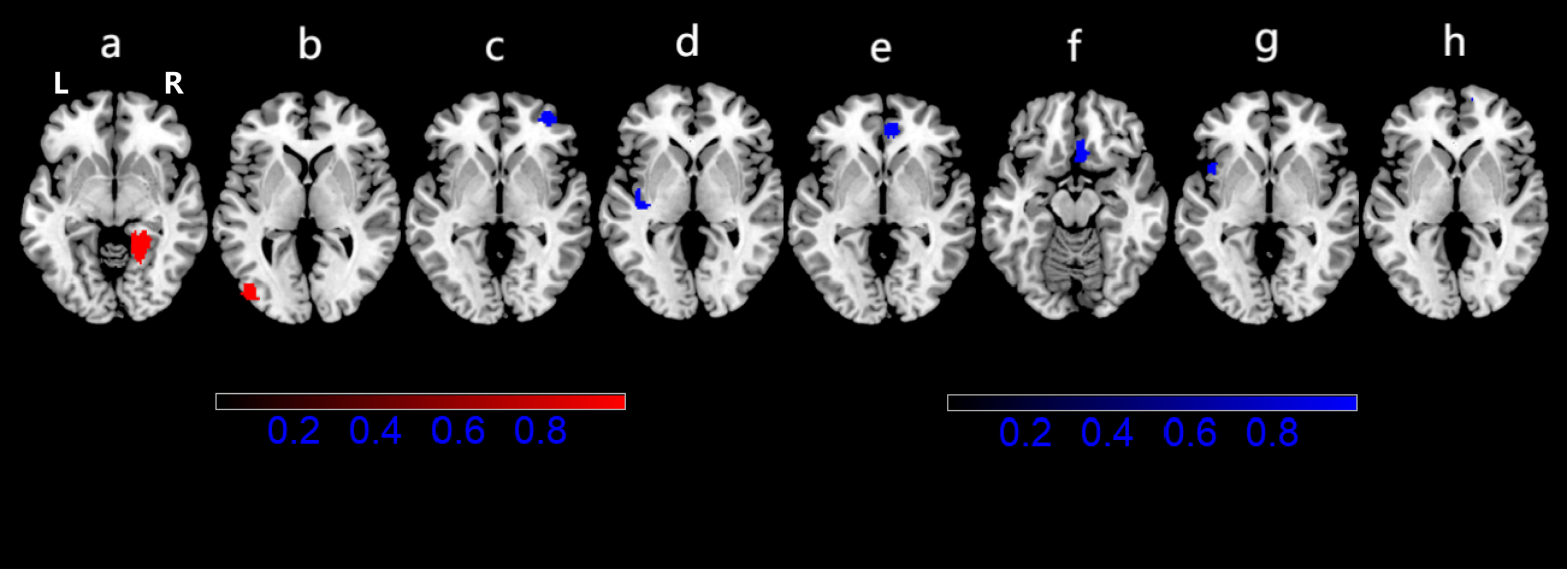
Areas with different GM volumes in smokers compared with non‐smokers in brain regions. Red clusters represent brain regions with increased GM, and blue clusters represent brain regions with decreased GM. (a) Right lingual gyrus; (b) left middle occipital gyrus; (c) right middle frontal gyrus (OFC); (d) left superior temporal gyrus; (e) right superior frontal gyrus SFG (ACC/OFC), BA10; (f) right superior frontal gyrus SFG (ACC/OFC), BA11; (g) left insula; and (h) corpus callosum.

Inclusion of 19 VBM studies (Table 1), meta‐analysis studies have shown that the brains of smokers exhibit elevated grey matter volumes in the right lingual gyrus and left middle occipital gyrus compared to non‐smokers. The right middle frontal gyrus (OFC), left superior temporal gyrus, right superior frontal gyrus SFG (ACC/OFC), left insula and corpus callosum had decreased grey matter (GM) volumes (Figure 2 and Table 2).

**TABLE 1:** Demographic and clinical characteristics and technical information of VBM studies included in the meta‐analysis.

Study (year of publication)	Sample (female)	Age (SD)	Smoking history (SD)	Cigarette/day (SD)	Pack‐years	FTND	FWHM (mm)	Threshold	Software
Gallinat (2006) [29]	Smokers 22 (12)	30.8 (7.5)	13.9 (7.3)	14.5 (9.2)	13.5 (13.0)	2.9 (1.7)	12	*p* < 0.05 corrected	SPM2
	Non‐smokers 23 (12)	30.3 (7.9)							
Almeida (2008) [5]	Smokers 39 (25)	75.0 (3.4)	59 (Na)	25 (Na)	Na	4	8	*p* < 0.005 uncorrected	SPM2
	Non‐smokers 39 (Na)	75.7 (3.2)							
Liao (2010) [30]	Smokers 44 (36)	28.1 (5.5)	10.4 (5.7)	20.3 (7.7)	Na	Na	12	*p* < 0.05 corrected	SPM5
	Non‐smokers 44 (34)	26.3 (5.8)							
Yu (2011) [9]	Smokers 16 (Na)	41.6 (5.5)	21.1 (3.9)	20.6 (7.4)	Na	7.19 (1.42)	8	*p* < 0.05 corrected	SPM5
	Non‐smokers 16 (Na)	39.2 (4.5)							
Zhang (2011) [31]	Smokers 48 (24)	31.4 (8.1)	12.8 (7.4)	20.9 (6.6)	12.9 (7.9)	5.4 (1.9)	8	*p* < 0.05 corrected	FSL
	Non‐smokers 48 (24)	31.1 (8.8)							
Morales (2012) [32]	Smokers 25 (13)	35.4 (1.8)	19 (Na)	14.1 (1.2)	11.5 (1.9)	3.8 (0.4)	8	*p* < 0.05 corrected	SPM8
	Non‐smokers 18 (8)	30.1 (2.2)							
Franklin (2014) [33]	Smokers 80 (39)	33.8 (Na)	14.1 (Na)	14.7 (Na)	10.5 (Na)	4.45 (Na)	8	*p* < 0.025 corrected	SPM8
	Non‐smokers 80 (39)	22.1 (Na)							
Fritz (2014) [7]	Smokers 315 (167)	44.1 (11.84)	26.8 (Na)	13.17 (6.99)	17.81 (12.25)	Na	12	*p* < 0.05 corrected	SPM8
	Non‐smokers 659 (416)	51.49 (14.45)							
Wang (2014) [34]	Smokers 22 (0)	22.48 (2.48)	4.95 (2.27)	11.90 (6.13)	3.10 (2.63)	Na	10	*p* < 0.05 corrected	SPM8
	Non‐smokers 20 (0)	21.80 (1.32)							
Peng (2017) [35]	Smokers 26 (0)	29.42 (4.43)	10.92 (Na)	16.15 (5.16)	8.77 (3.57)	5.15 (2.82)	6	*p* < 0.05 corrected	SPM8
	Non‐smokers 53 (0)	30.83 (5.18)							
Peng (2017) [35]	Smokers 27 (0)	32.26 (3.73)	12.7 (Na)	38.70 (8.36)	31.06 (7.40)	6.85 (2.18)	6	*p* < 0.05 corrected	SPM8
	Non‐smokers 53 (0)	30.83 (5.18)							
Wetherill (2015) [36]	Smokers 21 (9)	34.3 (9.4)	Na	14.1 (4.4)	10.2 (8.2)	4.2 (1.6)	8	*p* < 0.05 corrected	SPM8
	Non‐smokers 21 (7)	30.5 (8.8)							
Hanlon (2016) [37]	Smokers 58 (25)	31.7 (Na)	15.5 (Na)	16.2 (Na)	12.2 (Na)	4.6 (Na)	8	*p* < 0.01 corrected	SPM8
	Non‐smokers 60 (27)	29.7 (Na)							
Stoeckel (2016) [10]	Smokers 16 (4)	37.94 (11.61)	17.63 (10.49)	16.00 (4.84)	16.09 (12.17)	4.44 (2.16)	8	*p* < 0.05 corrected	SPM8
	Non‐smokers 16 (5)	34.19 (7.20)							
Bu (2016) [38]	Smokers 26 (0)	21.42 (1.73)	4.27 (2.44)	15.04 (4.82)	3.55 (2.97)	4.42 (2.20)	8	*p* < 0.05 corrected	SPM8
	Non‐smokers 26 (0)	20.58 (1.47)							
Peng (2018) [8]	Smokers 23 (0)	32.74 (4.34)	13.48 (Na)	18.07 (6.99)	15.93 (10.57)	3.43 (1.65)	6	*p* < 0.05 corrected	SPM8
	Non‐smokers 53 (0)	30.83 (5.18)							
Peng (2018) [8]	Smokers 30 (0)	30.70 (4.86)	11.83 (Na)	34.97 (7.31)	23.34 (13.33)	8.00 (1.02)	6	*p* < 0.05 corrected	SPM8
	Non‐smokers 53 (0)	30.83 (5.18)							
Qian (2019) [11]	Smokers 44 (Na)	39 (6.5)	18.9 (6.4)	23.6 (10.4)	Na	5.4 (2.4)	4	*p* < 0.01 corrected	SPM8
	Non‐smokers 41 (Na)	38.5 (7.4)							
Conti (2021) [6]	Smokers 28 (10)	28.1 (8.3)	12.0 (Na)	15.0 (4.5)	10.4 (8.1)	5.0 (1.5)	8	*p* < 0.05 corrected	SPM12
	Non‐smokers 24 (11)	28.5 (9.5)							
Ye (2020) [39]	Smokers 37 (7)	47.18 (7.22)	25.34 (9.23)	35.13 (10.7)	Na	8.89 (0.68)	8	*p* < 0.05 corrected	SPM8
	Non‐smokers 28 (7)	43 (9.62)							
Zhang (2023) [40]	Smokers 28 (0)	31.29 (5.56)	11.82 (5.77)	16.11 (8.35)	10.08 (7.95)	3.54 (2.03)	8	*p* < 0.01 corrected	SPM12
	Non‐smokers 28 (0)	31.68 (6.57)							

**TABLE 2:** Clusters of grey matter anomalies in cigarette smokers compared with healthy non‐smokers.

	Anatomical region	Peak MNI coordinates	Number of voxels	SDM‐Z value	*p*	Clusters' breakdown (number of voxels)	Jack‐knife sensitivity
Clusters of increased GM	R lingual gyrus, BA 37	20, −50, −6	602	1.375	0.000144482	R lingual gyrus, BA (17, 18, 19, 27, 30, 37) (189)	19 out of 21
						R fusiform gyrus, BA (30, 37) (111)	
						R parahippocampal gyrus, BA (27, 30, 37) (51)	
						(Undefined) (31)	
	L middle occipital gyrus, BA 19	−44, −82, 2	303	1.329	0.000201285	L middle occipital gyrus, BA (18, 19, 37, 39) (255)	19 out of 21
						(Undefined) (40)	
Clusters of decreased GM	R middle frontal gyrus (OFC)	36, 50, −2	182	−3.936	0.000015497	R middle frontal gyrus, BA (10, 46, 47) (122)	20 out of 21
						R anterior thalamic projections (48)	
	L superior temporal gyrus, BA 48	−40, −18, 0	187	−3.159	0.000650287	L insula, BA 48 (52)	19 out of 21
						L Heschl gyrus, BA 48 (50)	
						L superior temporal gyrus, BA 48 (41)	
						Corpus callosum (19)	
						(undefined), BA 48 (23)	
	R superior frontal gyrus, BA 10 (ACC)	8, 46, −2	162	−3.294	0.000407696	R superior frontal gyrus, BA 10 (78)	16 out of 21
						R anterior cingulate, BA (10, 11, 32) (83)	
	R superior frontal gyrus, BA 11 (OFC)	6, 24, −14	143	−3.402	0.000247717	R superior frontal gyrus, BA 11 (42)	20 out of 21
						Corpus callosum (40)	
						R gyrus rectus, BA 11 (19)	
	L insula, BA 48	−42,12,‐2	107	−3.034	0.001032174	L insula, BA 48 (49)	19 out of 21
						L inferior frontal gyrus, BA 48 (29)	

	Corpus callosum	12,62,0	42	−2.866	0.001677275	Corpus callosum (30)	20 out of 21
					R superior frontal gyrus, BA (10, 11) (12)	

### 3.2 Jackknife Sensitivity and Heterogeneity Analyses

Folding‐knife sensitivity analysis showed that GM volume differences in the right superior frontal gyrus (ACC) were reproducible in a combination of 16 datasets; GM volume differences in the right lingual gyrus, left middle occipital gyrus, left superior temporal gyrus and left insula were significant in a combination of 19 datasets, and GM volume differences in the right middle frontal gyrus (OFC), right superior frontal gyrus (OFC) and corpus callosum remained significant in a combination of 20 datasets; the above results are highly replicable.

### 3.3 Participant Characteristics

This study included 53 male smokers and 38 male non‐smokers. There was no significant difference in age and years of education between the two groups (*p* > 0.05) (Table 3).

**TABLE 3:** Demographics and clinical characteristics.

	Smokers (53)	Non‐smokers (38)	*t*	*p*
Age	34.58 ± 1.965	33.47 ± 6.805	0.759	0.450
Education	14.943 ± 1.965	15.39 ± 2.200	−1.028	0.307
FTND	4.02 ± 2.024			
Pack‐years	15.68 ± 10.189			

### 3.4 Results of rs‐FC Alterations Based on GM Volume Differential Brain Regions

**FIGURE 3:**
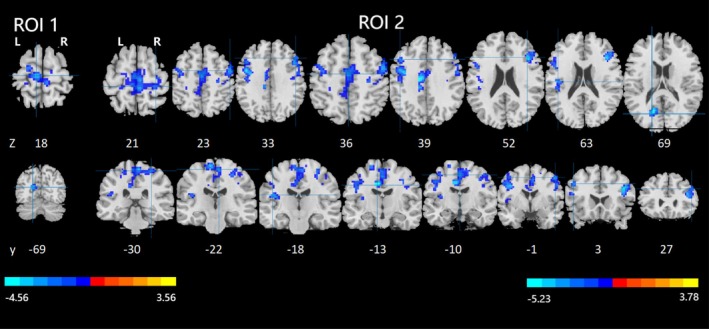
Altered resting‐state functional connectivity with the right lingual gyrus and right superior frontal gyrus (OFC). It shows brain regions with reduced functional connectivity to the right lingual gyrus and right superior frontal gyrus (OFC). Brain regions with increased functional connectivity to the right lingual gyrus and right superior frontal gyrus (OFC) were not identified. ROI 1: right lingual gyrus; ROI 2: right superior frontal gyrus (OFC).

**FIGURE 4:**
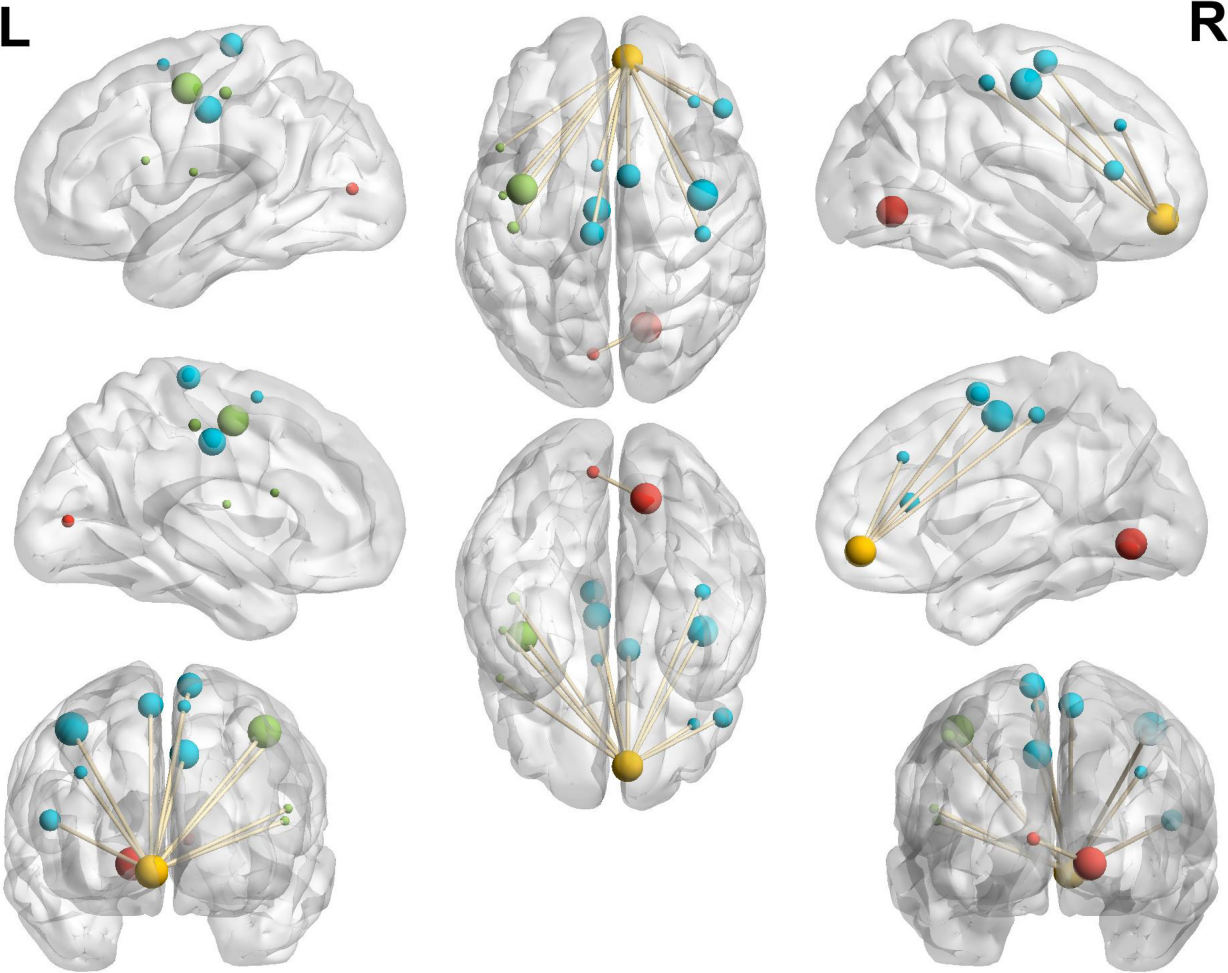
Functional connectivity map. Big red ball, right lingual gyrus; small red ball, left calcarine sulcus; yellow ball, right superior frontal gyrus (OFC); green balls, Cluster 1; blue balls, Cluster 2. Image created via BrainNetViewer.

Compared with non‐smokers, smokers had reduced functional connectivity from the right lingual gyrus (LING) to the left calcarine sulcus (CAL) and reduced functional connectivity from the right superior frontal gyrus (OFC) to the bilateral precentral gyrus (PreCG), the bilateral postcentral gyrus (PoCG), the left rolandic operculum (ROL), the left inferior frontal gyrus opercular part (IFGoperc), the left midcingulate cortex (MCC), the bilateral supplementary motor area (SMA), the left paracentral lobule (PCL), the right inferior frontal gyrus triangular part (IFGtriang) and the right middle frontal gyrus (MFG) (GRF‐corrected, P pixel < 0.001, P group < 0.05) (Table 4, Figure 3 and Figure 4).

**TABLE 4:** Brain regions with decreased functional connectivity between smokers and non‐smokers.

ROI	Cluster	Peak MNI coordinates (x, y, z)	Number of cluster voxels	*t*	Regions
R LING	Cluster 1	−18, −69, 18	58	−4.5627	L Cal
R OFC	Cluster 1	−45, 3, 33	191	−4.9444	L PreCG
		−45, −13, 39	55	−3.97324	L PoCG
		−44, −18, 21	47	−4.3334	L ROL
		−45, 21, 36	38	−4.45141	L IFGoperc
	Cluster 2	−9, −12, 36	154	−5.2328	L MCC
		50, −1, 52	185	−4.47779	R PreCG
		6, −21, 63	140	−4.36985	R SMA
		−9, −22, 69	120	−4.74525	L PCL
		39, 24, 23	97	−5.09464	R IFGtriang
		29, −30, 63	74	−4.08834	R PoCG
		48, 27, 33	67	−4.25526	R MFG
		0, −10, 52	65	−4.45108	L SMA

Abbreviations: CAL, left calcarine sulcus; IFGoperc, inferior frontal gyrus opercular part; IFGtriang, inferior frontal gyrus triangular part; L, left; LING, lingual gyrus; MCC, midcingulate cortex; MFG, middle frontal gyrus; OFC, superior frontal gyrus (OFC); PCL, paracentral lobule; PoCG, postcentral gyrus; PreCG, precentral gyrus; R, right; ROL, rolandic operculum; SMA, supplementary motor area.

### 3.5 Correlation Analysis

**FIGURE 5:**
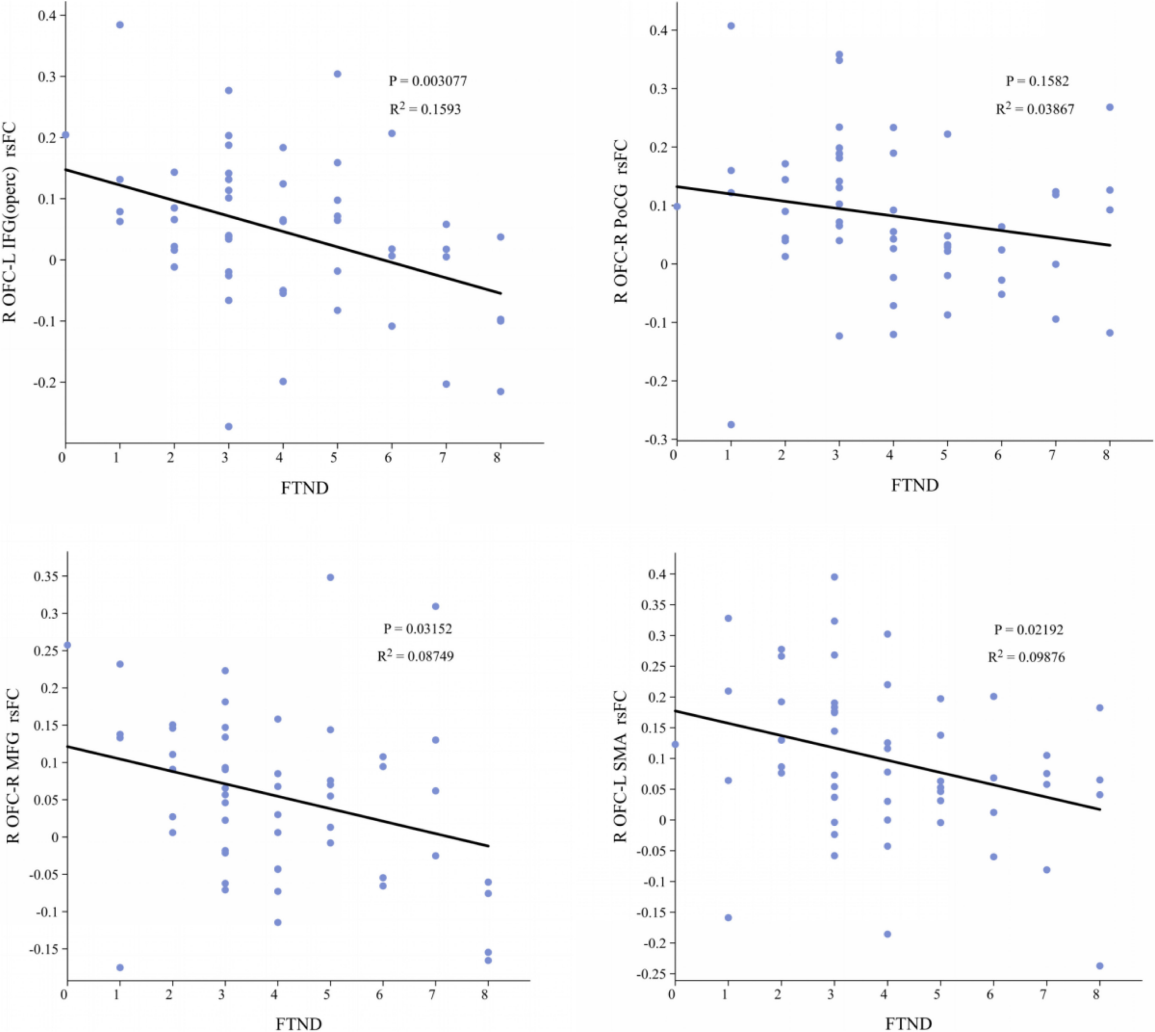
Correlations between R OFC–L IFGoperc/R MFG/R PoCG/L SMA and the FTND scores in smokers (uncorrected). FTND, Fagerström Test for Nicotine Dependence; L IFGoperc, left inferior frontal gyrus opercular part; L SMA, left supplementary motor area; R MFG, right middle frontal gyrus (MFG); R OFC, right superior frontal gyrus (OFC); R PoCG, right postcentral gyrus; rsFC, resting‐state functional connectivity. Image created via ChiPlot.

The results showed that reduced functional connectivity from the right superior frontal gyrus (OFC) to the left inferior frontal gyrus opercular part (IFGoperc), left paracentral lobule (PCL), right postcentral gyrus (PoCG), right middle frontal gyrus (MFG) and left supplementary motor area (SMA) was negatively correlated with FTND (*t* = −0.385, *p* = 0.004; *t* = −0.302, *p* = 0.028; *t* = −0.279, *p* = 0.043; *t* = −0.318, *p* = 0.02; *t* = −0.342, *p* = 0.012 uncorrected) (Figure 5). However, after Bonferroni correction, no significant correlation was obtained.

## 4 Discussion

In the present study, we investigated the functional connectivity changes in the grey matter volume of the brain of smoking addicts based on the brain regions that have been previously reported in the literature to undergo changes in grey matter volume. We found that the right lingual gyrus with elevated GM volume had reduced functional connectivity with the left calcarine sulcus; the right OFC with reduced GM volume had reduced functional connectivity with the bilateral PreCG, the bilateral PoCG, the left ROL, the left IFGoperc, the MCC, bilateral SMA, left PCL, right IFGtriang and right MFG.

The right lingual gyrus is considered to be an integral part of the occipital optic cortex, and an increase in its GM volume may be associated with an increase in neuroplasticity in this region [41]. Meanwhile, the orbitofrontal cortex (OFC) belongs to a subregion of the prefrontal cortex [42], and previous studies have found reduced GM volume or density in the prefrontal cortex [31, 43]. Differences in prefrontal cortical grey matter volume and density between smoking and non‐smoking groups may be related to the direct effects of cigarettes [44, 45] or cigarette cue exposure [46].

The calcarine sulcus and lingual gyrus belong to the visual attention area and play an important role in visual information integration and attentional processing [47]. Studies have found reduced static functional connectivity density in the calcarine sulcus in nicotine‐dependent individuals [48] and reduced connectivity in the visual (VIS) brain region containing the lingual gyrus in alcohol and cigarette addicts [49], suggesting that substance addiction can lead to reduced functional connectivity in functional areas related to visuospatial attention [50]. This may lead smokers to show top‐down attentional bias disorder and inhibitory attentional bias disorder to cigarette cues [51], which in turn may make it easier for them to gather information related to smoking, thus inducing craving and even smoking behaviour [23].

The executive control network (ECN) is a frontoparietal network that primarily includes the dorsolateral prefrontal cortex (DLPFC), inferior parietal lobule (IPL), middle frontal gyrus (MFG), ventral prefrontal cortex, middle temporal gyrus (MTG), supraparietal cortex (SPG), inferior frontal gyrus (IFG) and orbital frontal cortex (OFC) [52, 53]. The ECN is critical for cognitive control of working memory, thoughts, emotions, behaviours and relevant external information and is involved in inhibitory control and execution [54, 55]. The OFC is one of the important nodes of the ECN and plays a crucial role in decision making, reward assessment and preference selection [54, 55]. In addition, according to [56], addiction is associated with cognitive control dysfunction, and abnormal activity in the OFC causes cognitive control dysfunction. A growing number of researchers support the idea that failure of executive control can lead to addiction [57]. According to [53], a reduction in the strength of ECN connections in smokers was found by means of independent component analysis (ICA) and connection analysis. Individuals with Internet gaming disorder have also been found to exhibit reduced functional connectivity of the ECN during off‐task periods [58].

In this study, we found reduced functional connectivity of the OFC to the left IFGoperc, right IFGtri and right MFG. Consistent with previous findings: the FC between SFG and IFG was significantly reduced in TUD [53, 59]. In addition [3], reduced FC between L‐SFG and R‐IFG in the brains of temporary abstinence addicts and diminished functioning of the right MFG and SFG in patients with alcohol use disorders were also found [60]. The left IFG, BA 44, is a brain region involved in speech production [61], whereas the right IFG is strongly activated in stop‐signal tasks [62], and damage to the right IFG appears to disrupt performance in that stop‐signal task [63]. Thus, ineffectiveness of the right inferior frontal gyrus may be related to an impulse to produce too many movements [64]. MFGs are considered to be important nodes in the ECN [65] and are primarily involved in attentional control, inhibitory control and task switching, as well as other core cognitive functions [66]. Decreased connectivity of the OFC to the IFG and MFG indicates reduced communication and function within the ECN associated with nicotine use, which may lead to decreased cognitive control and greater susceptibility to stimulation in the face of external smoking‐related information, which in turn creates an urge to smoke leading to repetitive smoking behaviour.

There exists a view that area BA44 (the left IFG) projects to premotor cortical areas, such as supplementary motor areas [61]. In this study, we found reduced FC in the right OFC to bilateral PreCG, bilateral PoCG, bilateral SMA, left ROL and left paracentral lobule. The PreCG and PoCG and the SMA are associated with somatosensory and sensorimotor networks (SMN) [67, 68, 69]. Anatomically, the rolandic area is the cerebral cortex near the central sulcus, which includes the PreCG and PoCG. The paracentral lobule is a motor‐functional area located in the frontal anterior central gyrus, which interacts with somatosensory regions. There is an anatomical link between the DLPFC and the mOPFC [70]. Some researchers have found reduced information outflow from the right DLPFC to the left precentral/postcentral motor area and supplementary motor area (pre‐SMA) in betel nut addicts [67] and similar reductions in rsFC from the DLPFC to the precentral gyrus, postcentral gyrus and SMA in patients with online gaming disorder [71]. Studies have also found synchronous changes in ReHo in the paracentral lobule and SMA [22]. PreCG is associated with sensorimotor integration and motor impulses [72], and SMA structures play a role in automated behaviour and motor planning [73]. Substance use skills are central to drug acquisition and consumption behaviours and become highly automated with repeated practice [74]. These brain regions may be related to the sensorimotor aspects of smoking and can help inhibit smoking behaviour when refusal is required. Therefore, we suggest that a reduction in FC between the ECN and SMN may lead to reduced frontal activation, a reduced ability to inhibit smoking cravings and urges and difficulty in suppressing and generating smoking behaviours. It has been found that the prefrontal cortex, anterior cingulate cortex and primary motor and sensory cortex networks are less connected in people who have difficulty quitting smoking [75]. Strengthening the connectivity between the motor/sensory cortex and prefrontal cortex may be effective in inhibiting smoking cessation relapse [76].

The anteromedial SFG is anatomically connected to the anterior and middle cingulate cortex, which are key nodes of the cognitive control network and the default mode network (DMN) [77]. The anterior portion of the MCC (also referred to as the dorsal ACC in many studies) has been associated with cognitive controls such as conflict monitoring [78] error detection [79], response selection [80] and attentional control [81]. The MCC correlates reward outcome information from the OFC with action information from the posterior cingulate cortex, with output directed to premotor cortical areas [82]. Nicotine is known to bind to nicotinic acetylcholine receptors and is present in many areas contained in the ECN and DMN, particularly the frontal and cingulate cortex [83]. Previous studies have shown that when dopamine levels are reduced in individuals with tobacco use disorder (TUD), their inhibitory control is impaired and frontal activation is reduced [84]. In the present study, we found that the reduced FC from the OFC to the MCC may indicate that reduced dopamine levels and diminished frontal lobe activation in TUD patients may lead to a reduction in the output of reward information from the OFC to the MCC, which is exported to the premotor cortex and may cause spontaneous smoking behaviour. However, the causal relationship between the MCC and premotor cortex still needs to be verified by using methods such as dynamic causal modelling (DCM).

Correlation analyses showed that reduced functional connectivity from the right superior frontal gyrus (OFC) to the left IFGoperc, the left paracentral lobule, the right PreCG, the right MFG and the left SMA was negatively correlated with FTND. This suggests that the more severe the nicotine dependence, the weaker the functional connectivity between the above brain regions. However, this finding did not survive correction for multiple comparisons, which may be attributable to the relatively small sample size of the study.

There are some limitations to this study. First, the smoking subjects were all male, and female smokers were not included. It has been shown that male smokers are more sensitive to the enhancing effects of tobacco than female smokers [25]. Therefore, the results of this study are not applicable to all populations. Second, the sample size of this study was small, and there may be racial and geographic differences from the findings of other researchers. Further, the present study was a cross‐sectional study, and future longitudinal studies should be conducted to confirm and supplement the findings of the present study on changes in functional brain connectivity.

## 5 Conclusion

Studies have shown that smoking causes significant changes in brain structure and functional connectivity. Corresponding functional connectivity changes exist in parts of the brain where grey matter volume is altered in smokers. Reduced functional connectivity of the executive control network with regions of the visual attention network and the sensorimotor network suggests impaired neural communication between the executive control network and them, which may contribute to difficulties in quitting smoking. However, further longitudinal studies are needed to validate this idea. The study also found a correlation between these changes and the severity of nicotine dependence (uncorrected) and may lead to difficulties in quitting. The findings emphasize the importance of developing more effective smoking cessation interventions that target these neurobiological changes.

## Author Contributions

**Hui Zhang:** writing – original draft, visualization, validation, methodology, survey, formal analysis, data management, conceptualization. **Jiawen Tian:** writing – original draft, methodology, formal analysis, data management. **Xinyu Wang:** writing – original draft, methodology, formal analysis, data management. **Hongyu Zhang:** writing – original draft, methodology, formal analysis, data management. **Longyao Ma:** writing – commenting and resources, methodology. **Bohui Mei:** writing – commenting and resources, methodology. **Mengzhe Zhang:** writing – commenting and resources, methodology. **Qingqing Lv:** writing – commenting. **Yarui Wei:** methodology, resources. **Shaoqiang Han:** methodology, resources. **Yong Zhang:** writing – review, supervision, resources, methodology, funding acquisition, conceptualization. All authors provided editorial comments and approved the final version.

## Ethics Statement

This study involving human participants was conducted in accordance with the ethical standards of the local medical ethics committee of the First Affiliated Hospital of Zhengzhou University. Written informed consent was obtained from all participants before their involvement in the study.

## Conflicts of Interest

The authors declare no conflicts of interest.

## Data Availability

The datasets used in this study are not publicly accessible due to participant privacy concerns. Requests for access to the datasets should be directed to Yong Zhang.
